# Immunization against Lamb Haemonchosis with a Recombinant Somatic Antigen of *Haemonchus contortus* (rHcp26/23)

**DOI:** 10.4061/2010/852146

**Published:** 2010-06-29

**Authors:** Leticia García-Coiradas, Francisco Angulo-Cubillán, Basilio Valladares, Enrique Martínez, Concepción de la Fuente, José María Alunda, Montserrat Cuquerella

**Affiliations:** ^1^Departamento de Sanidad Animal, Facultad de Veterinaria, Universidad Complutense de Madrid, 28040 Madrid, Spain; ^2^Departamento de Enfermedades Transmisibles, Facultad de Veterinaria, Universidad del Zulia, 4005-A Maracaibo, Venezuela; ^3^Departamento de Parasitología, Facultad de Farmacia, Universidad de La Laguna, 38271 Tenerife, Spain

## Abstract

Haemonchosis, caused by the abomasal nematode *Haemonchus contortus*, is a common parasitic disease of sheep. Our previous results showed that a soluble fraction from adult stages of the nematode (p26/23) induced partial protection against challenge. Recombinant DNA technology was applied to obtain a synthetic protein (rHcp26/23). Immunological assays (ELISA, Western blotting, and immunolocalization), using sera from lambs immunized with p26/23, confirmed the identity of the recombinant protein and demonstrated that the synthetic protein is equivalent to the purified protein employed in the previous immunoprophylaxis studies. Vaccination of lambs with 300 *μ*g of rHcp26/23 and Freund's adjuvant elicited a notable specific antibody response. Immunization did not induce any significant protection after challenge with 16000 infective larvae of *H. contortus*, and comparable values for parasite faecal egg output, packed cell volume, and abomasal parasite burdens were found in vaccinated and control animals.

## 1. Introduction

*Haemonchus contortus *(Trichostrongylidae, Nematoda) is the etiological agent of haemonchosis, a worldwide distributed parasitic disease affecting small ruminants, particularly sheep and goats. Infections by this helminth cause digestive disturbances such as inappetence, alterations of energy and protein metabolism, and anemia accompanied, in severe cases, by hypoproteinemia and oedema [1]. The control of the disease has been based, almost exclusively, on the use of anthelmintics. However, their massive and indiscriminate use has led to the appearance of parasite isolates with anthelmintic resistance to the main antiparasitic drug groups [2]. This situation has triggered efforts towards the exploration of potentially immunoprotective antigens. Among the most promising are H11, H-gal-GP, some excretory-secretory (ES) antigens, and the somatic antigen p26/23 [3–8]. Results obtained in the different vaccination trials have varied, some of them achieving notable protection levels from 32.2–90% reductions in parasite egg shedding and 61–78% reductions in worm populations in the abomasa of vaccinated lambs. Most investigational endeavours have concentrated on parasite antigens not detected by infected hosts, the so-called “hidden antigens” (HAGs) normally present in the parasite's gut [9, 10]. This approach has been successfully used to vaccinate against cattle ticks with Bm86 [11]. In spite of the potential advantages of this approach [12], the protection levels achieved with HAGs are dependent on the revaccination of animals; therefore labour costs and management methods of small ruminants render this approach less convenient. Conversely, exposed antigens (“natural antigens”, NAGs), although subjected to natural selection, could be used in the absence of re-vaccination because exposure to the parasite would act as a natural booster for hosts. Our group has already demonstrated that a protein fraction obtained from adult worms, p26/23, was immunoprotective in 3.5–5-month-old lambs challenged with *H. contortus * [4, 13]. Recently, the content of this fraction has been analyzed, and the major protein present has been purified, immunolocalized, and partially sequenced [14]. In the present paper we present the cloning and expression of the recombinant protein, rHcp26/23. In addition, a pilot immunization experiment against *H. contortus* challenge has been carried out.

## 2. Material and Methods

### 2.1. Helminths

Adult *H. contortus* were recovered from the abomasa of monospecifically infected lambs 2-3 months of age with 12000 third stage larvae (L3) of a parasite isolate originally obtained from Merck, Sharp & Dohme, Spain in 1987 and maintained in our facilities by serial passage in donor lambs. Animals were fed with commercial pelleted food (Superfeed), hay, and water *ad libitum*. Forty-two days after infection, lambs were slaughtered, and adult helminths were recovered from the abomasal content and mucosa. Helminths were extensively washed with 150 mM PBS (pH 7.4) containing protease inhibitors, 1 mM phenyl metasulfonile fluoride (Fluka), and Ethylenediamine tetraacetic acid (EDTA) (Sigma) at 4°C. Helminths were preserved at −80°C until used. L3 were obtained by fecal culture (26°C, 10 days, and >80% relative humidity), baermannization, and partial purification on filter paper. Recovered larvae were washed with PBS and preserved at 4°C for infection purposes or at −80°C for biochemical and immunological studies.

### 2.2. Purification of p26/23 from H. contortus

Adult worms, largely females, were subjected to freezing and thawing cycles (−20°C, room temperature), homogenized, and centrifuged at 30000 × g, 4°C for 30 min to obtain the supernatant (Adult Soluble Extract, ASE). Purification of p26/23 involved the sequential use of affinity chromatography with S-Hexyl Glutathione and reverse-phase chromatography (Vydac 214TP5415 column) [14, 15]. Proteins were analyzed by 15% polyacrylamide gel electrophoresis with 1% sodium dodecyl sulphate (Merck) and 5% mercaptoethanol (Merck) (SDS-PAGE). Proteins were electrotransferred to nylon membranes (Immobilon P, Millipore). Pooled sera from lambs immunized with the fraction p26/23 [13] were used in the Western blotting (WB) to identify the protein. Membrane strips containing p26/23 were excised, the protein was eluted [16], and N-terminal sequence was determined (Centro de Investigaciones Biológicas, CIB, CSIC, Madrid). Larval soluble extract (LSE), for SDS-PAGE and WB, was obtained in the same manner as ASE. Protein concentration was determined by a modified Bradford method (Bio Rad protein assay).

### 2.3. Sera

Rabbit hyperimmune sera against purified p26/23 were obtained from 2 New Zealand X California Giant rabbits, 2 months of age. Animals received 3 intramuscular doses of 20 *μ*g each of the protein, at 14-day intervals. First injection was administered with Freund Complete Adjuvant (Sigma) and the next two with Freund Incomplete Adjuvant (Sigma). To obtain antisera against the recombinant protein a similar protocol was used, although 100 *μ*g were used on each injection. Sera from lambs immunized with the fraction containing native p26/23 came from a previous experiment [4, 13].

### 2.4. Nucleic Acid Extraction and Amplification of p26/23 Coding Sequence

Adult *H. contortus *(10–15) were homogenized in 1 mL TRIzol (Gibco BRL) [17]. Recovered RNA in the aqueous phase was precipitated with 0.5 mL isopropanol for 10 min at room temperature and centrifuged at 12000 × g (10 min, 4°C). Precipitate was washed with ethanol for 5 min at 7500 × g and 4°C and resuspended in RNAse-free water. Extract was subjected to treatment with DNAse (Dnase I Rnase-free, Roche) [18]. RT-PCR was carried out in two independent steps: synthesis of cDNA (1st Strand cDNA Synthesis Kit, AMV, Roche) using hexanucleotide random primers in 40 *μ*L final volume and amplification by PCR following the procedures described below. The partial N-terminal amino acid sequence of the protein p26/23 purified from adult *H. contortus* [14] showed 85% identity with the hypothetical protein HCC00515 from *H. contortus* (NEMBASE), deduced on the nucleotide sequence from a cDNA library. Therefore, the available N-terminus sequence of this protein and the nucleotide sequence of HCC00515 were used as template to design two primers with BamHI and HindIII restriction targets: FBamHI (5′  GGA TCC GCA GGA CTG TTC GCA CAT  3′) and RHindIII (5′  AAG CTT TCA GTC TTT CGC GGA CTT G  3′). Reaction mixture included 1 *μ*L cDNA, 0.2 mM of each of deoxynucleoside triphosphates (dATP, dCTP, dGTP, and dTTP) (Roche), 50 mM KCl, 2.5 mM MgCl_2 _, 3 ng/*μ*L of each primer, 5 IU of Taq DNA polymerase (Roche), and 10 mM Tris HCl, pH 8.3, in a final volume of 50 *μ*L. The PCR amplification included denaturalization for 3 min at 94°C, followed by 30 cycles (95°C, 1 min) with one annealing-elongation step at 71°C for 1 min and, finally, an elongation step at 72°C for 10 min. PCR was carried out in a PTC-100 (MJ Research Inc.). The resulting fragment was cloned in the vector pGEM-T (Promega), and the construct was used to transform *Escherichia coli* XL2-blue [18]. Positive bacterial colonies were identified by PCR (94°C, 1 min; 54°C, 1 min; 72°C, 1 min), followed by 7 min, 72°C treatment, employing the primers SP6 (5′  ATT TAG GTG ACA CTA TAG AA  3′) and T7 (5′  TAA TAC GAC TCA CTA TAG GG  3′). minipreps were prepared with PCR positive colonies (QIAprep Spin miniprep kit (250) (Qiagen)).

### 2.5. Expression and Purification of Recombinant p26/23 (rHcp26723)

The insert was cloned in the expression vector pQE30 (QIAexpress vector, Qiagen), and the construct was employed to transform *E. coli* M15 (PREP 4) (Qiagen). Positive bacterial colonies were identified by PCR employing the primers FpQE (5′  GAA TTC ATT AAA GAG GAG AAA  3′), for the plasmid and R (5′  TCA GTC TTT CGC GGA CTT G  3′), for the insert. The nucleotide sequence of PCR products and the positive bacterial clones in *E. coli* XL2-blue and M15 were determined by the Department of Genetics (Faculty of Veterinary Science, UCM, Madrid). The expression of the recombinant protein (rHcp26/23) was carried out with a PCR positive clone of *E. coli* cultured in LB broth medium with 100 *μ*g/mL ampicillin (Roche) and 25 *μ*g/mL kanamycin (Sigma) at 37°C. Cultures were induced with 0.02–2 mM isopropyl-*β*-thiogalactopyranoside (IPTG) (Roche) treatment. Cell pellets from cultures were resuspended, and protein was solubilized in both denaturing and nondenaturing conditions. In the purification under denaturing conditions, pellets were incubated in lysis buffer with 8 mM urea centrifuged, and supernatants, recovered. The process was repeated four times. The recombinant His_6 _tagged p26/23 was purified in 10 × 1 cm columns (Bio Rad) of Ni-NTA agarose (Qiagen). Solubilization under nondenaturing conditions was carried out by resuspending cell pellets in a buffer with 20 mM imidazole at pH 8.0. Cells were disrupted by sonication, and the purification of the recombinant protein was carried out with the same column as above and eluted with 250 mM imidazole. Proteins obtained from both purification methods were dialyzed against PBS and lyophilized until use. Recombinant proteins were analyzed by SDS-PAGE and WB using pooled sera from vaccinated lambs with the fraction p26/23.

### 2.6. Immunolocalization of p26/23 in H. contortus

Immunolocalization of p26/23 in adult *H. contortus* was carried out following the methods described in [14]. Sections were washed with PBS, blocked with 5% foetal bovine serum (Sigma) in PBS for 1 h at room temperature, and incubated with rabbit anti-p26/23 and pooled anti-rHcp26/23 sera (1 : 1000 in PBS). The slides were mounted in Crystal/ Mount aqueous-dry mounting medium (Biomeda) and examined under an Olympus BX-60 fluorescence microscope.

### 2.7. Lambs Vaccination with Fraction p26/23

#### 2.7.1. Lambs and Experimental Design

Twenty-four, 3-month old female helminth-free lambs (Manchego breed) were obtained from a local producer (Toledo). Animals were kept at isolation stables from our facilities, clinically monitored along the experiment, and the conditions were approved by the Madrid Veterinary Faculty Committee for Animal Experimentation. Lambs were distributed in a stratified manner (live weight) onto 5 experimental groups. Group 1 animals (no. 1, 2, 3, 4, and 5) were immunized with rHcp26/23 purified under nondenaturing conditions; Group 2 animals (no. 6, 7, 8, 9, and 10) received denatured rHcp26/23; Group 3 animals (no. 11, 12, 13, 14, and 15) received only adjuvant; Group 4 animals (no. 16, 17, 18, 19, and 20) were the unvaccinated, challenged control, Group 5 animals (no. 21, 22, 23, and 24) were the unvaccinated and unchallenged negative control. Lambs from Groups 1 and 2 received immunizing injections (intramuscular and subcutaneous in the groin and hind legs) on days 0, 14, and 28. The first injection (100 *μ*g recombinant protein) was administered in 1 mL Freund's Complete Adjuvant; the second and third injections were administered in 1 mL Freund's Incomplete Adjuvant. On the same days lambs from Group 3 received only adjuvant. On day 42, animals from Groups 1–4 were challenged with 16000 L3 of *H. contortus* by means of bucoesophagic catheter.

#### 2.7.2. Blood Sampling, Parasitological, Biopathological, and Immunological Determinations

Coproscopical analyses were carried with a modified McMaster technique [19]. Along the experiment blood samples were obtained by jugular venipuncture in evacuated tubes (Vacutainer) every 14 days. Packed cell volume (PCV) and haemoglobin concentration were determined with standard laboratory techniques. Serum-specific antibody response was determined by ELISA and Western blot. In the ELISA, microplate wells (Nunc) were coated with 5 *μ*g/mL (ASE, LSE) or 1 *μ*g/mL (rHcp26/23). Individual lambs' sera were diluted 1 : 400 in PBS, and second antibody was alkaline phosphatase-labelled anti-sheep IgG (Sigma) 1 : 30000 diluted. Absorbance was read at 405 nm. Western blots were carried out with ovine (1/100 dilution) or rabbit sera (1/1000 dilution) for 2 h at 37°C. Secondary antibody was peroxidase-labelled mouse monoclonal (clone GT-34) anti-sheep IgG (Sigma) and employed at 1/1000 dilution for 1 h, 37°C. Peripheral lymphocyte response was determined following previously described techniques with slight modifications [13, 20]. Briefly, blood samples in 1% heparin (Rovi) obtained from the experimental animals were diluted (1 : 1) in RPMI 1640 medium (Bio-Whitakker). The lymphocyte enriched fraction was obtained from the interface, after centrifugation (800 × g) for 25 min, of 4 mL Ficoll-Paque Plus (Amersham Biosciences) with diluted blood on it. Viable cells (2.5 × 10^4^) were resuspended in RPMI supplemented with 10% foetal bovine serum (Sigma) and 1% penicillin-streptomycin mixture (Bio-Whittaker). Antigens were employed at 5 *μ*g/mL (ASE) or 1 *μ*g/mL (rHcp26/23). Cultures were kept for 5 days at 37°C in a 95% air/5% CO_2_ atmosphere. Concanavalin (Amersham) was used as positive internal control (3 days at 2.5 *μ*g/mL). Cultures were pulsed with 2 *μ*Ci/mL of methyl-H^3^ thymidine (Amersham) 18 hours before harvest. Results were expressed as stimulation indexes (SI) [mean counts per min (cpm) of the triplicate stimulated cultures/mean cpm of the triplicate nonstimulated cultures]. On day 42 post challenge, animals were slaughtered at a local abattoir (Getafe, Madrid), abomasa removed, and taken to the laboratory under refrigeration. Individual abomasa were opened and the adult helminths in the content and the mucosa subsequently washed in cold PBS. A 10% aliquot of total recovered helminths was fixed in 5% buffered formalin and the worms present, counted (males, females). To determine the parasite dry weight 40 male and 40 female *H. contortus* were weighed, kept at 100°C, until they reached a constant weight. Parasite length was measured in 40 male and female helminths employing a transilluminator and curvimeter [21].

### 2.8. Statistical Analysis

To normalize variance worm burdens were log(*x* + 1) transformed, and a nonparametric test (two-tailed Mann-Whitney) was employed to calculate the statistically significant differences. Average comparisons were carried out to estimate the effect of vaccination on different days (IgG, faecal egg output) with a two-tailed Student *t* test. The level of significance was established at *P* < .01.

## 3. Results

### 3.1. Expression and Purification of rHcp26/23

**Figure 1:**
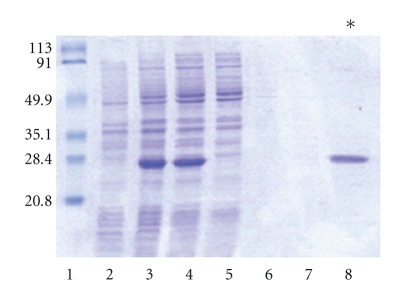
SDS-polyacrylamide gel of Coomassie blue stained fractions during purification under nondenaturing conditions of rHcp26/23. Lane 1: MW markers in kDa; 2: uninduced *E. coli* culture; 3: induced *E. coli* culture; 4: starting sample; 5: unbound fraction; 6: eluate with buffer I (without imidazole); 7: eluate with 20 mM imidazole; 8: eluate with 250 mM imidazole.(*) purified rHcp26/23.

The optimal overexpression of rHcp26/23 in the pQE30/*E. coli* M15 system was achieved after induction of bacterial cultures with 0.02 mM IPTG for 2 h (not shown). Electrophoretic analysis of supernatants and sediments obtained from the cultures of transformed *E. coli* showed that most of the protein was solubilized in the first washing with 8 M urea. Affinity chromatography allowed the purification of the recombinant protein between pH 5.9 and 4.5. Similarly, extraction under nondenaturing conditions rendered, after affinity chromatography, a single band, as assessed by SDS-PAGE (Figure 1, lane 8).

### 3.2. Cross-Reactivity between the Native (P26/23) and the Recombinant Protein (rHcp26/23)

**Figure 2:**
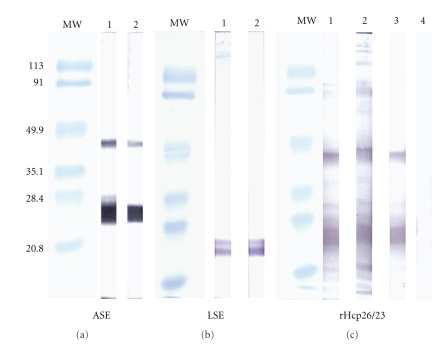
Western blots of adult (ASE) (a) and third-stage larvae (LSE) (b) soluble extracts of *H. contortus* and rHcp26/23 (c). Proteins were subjected to SDS-PAGE, transferred to nylon membranes, and probed with rabbit hyperimmune sera against native p26/23 (Lanes 1) and rHcp26/23 (Lanes 2), pooled sera from lambs vaccinated with p26/23 (Lane 3), and negative sheep serum (Lane 4). MW: Molecular weight markers in kDa.

**Figure 3:**
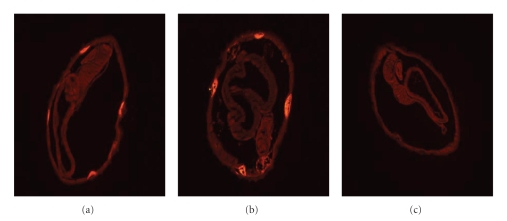
Immunolocalization of p26/23 in histological sections of adult *H. contortus*. Fluorescence was detected with antinative p26/23 hyperimmune rabbit serum and an anti-rHcp26/23 hyperimmune rabbit serum. Antirabbit Cy3-conjugated was the secondary antibody. Sections of adult *H. contortus* showed p26/23 recognition in the hypodermic chords of the nematode with anti-p26/23 (a) and anti-rHcp26/23 (b). Section incubated with normal rabbit serum (c).

WB analysis of rHcp26/23, and soluble extracts from both infective larvae (LSE) and adult stages (ASE) of *H. contortus* with sera from rabbit antinative p26/23, rabbit anti-rHcp26/23, and lamb anti-p26/23 fraction, showed comparable reactivity patterns irrespective of the antigen source (LSE, ASE). A clear band, ca. 24–26 kDa, was developed in both stages of the helminth, using the serum against the native p26/23 (Figures 2(a) and 2(b), lane 1) or the anti-rHcp26/23 (Figures 2(a) and 2(b), lane 2). It was also observed that the recombinant protein was recognized by the sera from vaccinated lambs (p26/23) (Figure 2(c), lane 3). Some reactivity was found at ca. 46 kDa, particularly when ASE (Figure 2(a)) and rHcp26/23 (Figure 2(c)) were probed with immune sera. Antigenic similarity between p26/23 and rHcp26/23 was confirmed by immunolocalization studies. Both hyperimmune sera (anti-p26/23 and anti-rHcp26/23), but not the serum from nonimmunized rabbit, specifically reacted against the hypodermic chords of adult *H. contortus* sections (Figure 3).

### 3.3. Immunization Trial

**Figure 4:**
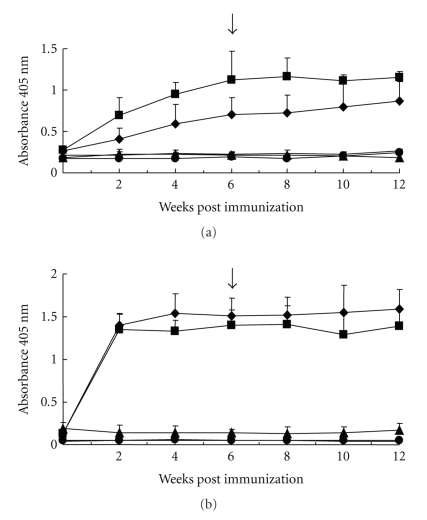
Serum-specific IgG response, determined by ELISA, of lambs along immunization with rHcp26/23 and challenge with 16000 third-stage larvae of *H. contortus*, against rHcp26/23 (a) and adult soluble extract of the parasite (ASE). G1 (■): lambs (*n* = 5) immunized with 3 doses (100 *μ*g each) of recombinant p26/23 (rHcp26/23) purified under nondenaturing conditions and challenged; G2 (♦): lambs vaccinated with 3 doses of rHcp26/23 purified under denaturing conditions and challenged; G3 (▲): lambs receiving adjuvant injections and challenged; G4 (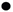): unvaccinated and challenged lambs. Arrow: day of experimental challenge. Values are means ± standard deviation of three determinations.

**Figure 5:**
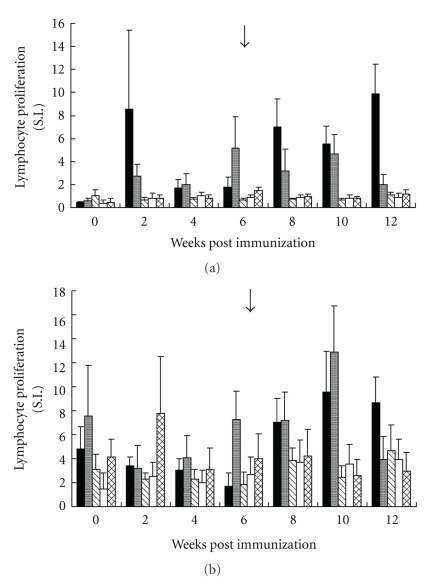
Peripheral lymphoproliferative response of lambs along the experimental immunization and challenge with 16000 third stage larvae of *H. contortus *in the presence of 1 *μ*g/mL recombinant protein (rHcp26/23) (a) and 5 *μ*g/mL adult soluble extract (ASE) of the helminth (b). SI: Stimulation index. Arrow: day of challenge. Values are the means ± standard deviation of three determinations. G1: solid bars; G2: grey bars; G3: stripped bars; G4: white bars; G5: woven bars.

**Figure 6:**
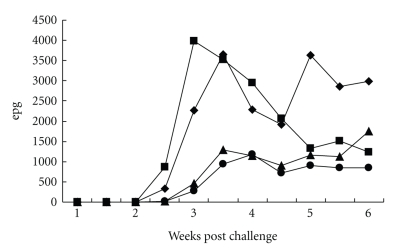
Average faecal egg output of experimental lambs (eggs per gram (epg)) along the challenge infection with 16000 third stage larvae of *H. contortus* administered by buco-oesophagic catheter. Symbols as in Figure 4.

Immunization with the recombinant protein elicited a strong specific IgG systemic response against the recombinant protein after the first immunizing injection (Figure 4(a)). Interestingly, immunized animals with the recombinant protein also displayed a strong response in ELISA against ASE of *H. contortus* (Figure 4(b)). Comparable results were observed in the Western blots with individual lambs' sera from the patent period of the infection (not shown). Similarly, immunization induced significantly higher lymphoproliferative response of lambs after challenge against the recombinant protein (Figure 5(a)) and soluble extracts of *H. contortus* (Figure 5(b)) and larval extracts of the helminth (not shown). Immunized lambs (G1 and G2) showed a slight shortening of the duration of prepatent period (18 days) when compared to nonvaccinated and challenged animals (G3 and G4). However, average data of parasite eggs faecal shedding from immunized animals were higher than those found in the control groups (Figure 6). These differences were not significant given the high variation found within each experimental group. All challenged animals displayed a transient fall of both PCV and haemoglobin concentration (not shown), during the prepatent infection and without differences among groups. Adult helminths were recovered from all challenged animals at necropsy (Table 1). The recovery rate (%) of the infective dose administered was low and no evidence of protection afforded by immunization with rHcp26/23 purified under nondenaturing (Group 1) or denaturing (Group 2) conditions was obtained (*P* > .5).

## 4. Discussion

A number of potentially protective HAGSs of *H. contortus* have been cloned and expressed, among them H11 and H-gal-GP [7, 10]. Few attempts have been made to clone NAGS from this helminth species, except for the expression of secretory/excretory proteins, particularly ES24 [22]. The present paper describes the cloning and prokaryotic expression (*E. coli*) of the* H. contortus* somatic antigen p26/23, a protein showing a notable immunoprophylactic value against lamb haemonchosis [4, 13]. The protocol used for the cloning and expression was very efficient, and purified histidine-tagged rHcp26/23 was obtained both in nondenaturing and denaturing conditions. The approximate MW of rHcp26/23 has a comparable value (23.16 kDa) to that reported for the native protein (24 kDa) [14]. No differences were observed in electrophoretic motility between the protein purified under denaturing and nondenaturing conditions. The expressed protein (rHcp26/23) was recognised in WB by sera from vaccinated lambs with p26/23 [4] and also by rabbit anti-p26/23 hyperimmune serum. Moreover, sera raised against the recombinant protein showed a similar recognition of the purified native protein. These results suggest that the recombinant protein corresponds to the native peptide present in the immunoprotective fraction described by these authors. In the WB, rabbit and lamb immune sera revealed the expected MW (ca. 23-24 kDa), and a minor recognition was seen around 46 kDa. A comparable finding was obtained in our previous work with the protective fraction [4] and the purified native protein [14]. Given the purification method (elution from membrane-bound proteins) no possible contaminants of twofold MW could be present. These results suggest that p26/23 might spontaneously dimerize thus displaying two reactive bands in WB. The immune recognition pattern found in nematode sections constitutes an additional evidence of the similarity of the native protein and rHcp26/23. In addition, it reinforces the conclusion that p26/23 is not related to ES24 since the latter is produced in the oesophagus and excreted/secreted during *H. contortus* feeding [22].

In spite of the strong immune response (IgG and lymphoproliferative response) elicited by the immunization of lambs with the recombinant protein (rHcp26/23) no protection against the *H. contortus* challenge was found. Actually no significant differences were found in helminth burdens in abomasum at the end of the experiment. In addition, no immunization-related variations were found in the epg and biopathological parameters determined (PCV, Hb). The results with the recombinant protein sharply contrast with those obtained with the fraction containing p26/23 [4] where vaccination of younger than 5-month-old lambs elicited a reduction over 60% in abomasal worm burdens and epg counts. 

Our results are in line with those obtained in most vaccination trials against haemonchosis with recombinant antigens (H11, H-gal-GP, and ES) whereas the native counterparts were able to induce significant levels of protection against challenge [7, 23]. A number of reasons have been invoked; among them the inadequate renaturalization of the recombinant proteins and glycosylation of the protective antigens [24–26]. The protein expressed, p26/23, apparently is not glycosylated [27]. Present experiment was carried out with a very similar design to that used with the native fraction with a significant protective effect [4]. Moreover, the results obtained in ELISA and lymphocyte proliferation tests rule out the inadequacy of the immunizing protocol followed. It has been suggested that recombinant expression could be an inadequate strategy to helminth vaccination. However, some encouraging results have been obtained with recombinant galectin in adult goats [28]. In many vaccination trials with unsuccessful results it is not known whether the native parasite antigen itself could induce protection or the relative importance of its epitopes [29]. In our case the native p26/23 elicited significant protection in similar conditions [4]. Given the economic importance of haemonchosis, further research is needed. In particular, the protein should be expressed in eukaryotic systems, critical epitopes determined, and alternative immunization strategies explored.

## Figures and Tables

**Table 1:** Number of adult *Haemonchus contortus* (females, males, total helminths) and recovery rate (as percentage of infective dose administered) recovered from the abomasa of challenged lambs at the end of the experiment. Helminth counts were log(*x* + 1) transformed. Numbers within brackets represent the parasite biomass (mg) determined. Data are average ± standard deviation.

Group	Nr. Animal	Females	Males	Adults	% Recovery
G1	1	3.17	3.01	3.4	15.81
	2	2.97	2.8	3.201	9.93
	3	1.78	1.32	1.9	0.5
	4	3.25	3.28	3.57	23.43
	5	3.2	3.26	3.53	21.56
		3.07 ± 2.8	3.03 ± 2.9	3.35 ± 3.2	14.25 ± 9.3
		(252.7 ± 145)	(124.37 ± 86)	((377.07 ± 253.88)	

G2	6	1.85	1.7	2.08	0.75
	7	2.84	2.23	2.94	5.43
	8	3.19	3.13	3.46	18.18
	9	2.04	1.7	2.2	1
	10	3.71	3.66	3.98	61.06
		3.18 ± 3.3	3.09 ± 3.28	3.44 ± 3.61	17.28 ± 25.47
		(377 ± 481.64)	(100.65 ± 121.3)	(496.43 ± 596)	

G3	11	2.51	2.17	2.68	3
	12	2.91	2.79	3.15	9
	13	2.66	2.5	2.89	4.87
	14	2.54	2.14	2.69	3.06
	15	3.193	3.13	3.46	18.25
		2.84 ± 2.71	2.71 ± 2.70	3.08 ± 3.01	7.63 ± 6.41
		(191.95 ± 127.0)	(53.95 ± 48.99)	(245.9 ± 168.5)	

G4	16	3.32	3.28	3.6	25.25
	17	2.99	2.91	3.25	11.31
	18	2.55	2.39	2.78	3.81
	20	3.12	2.919	3.33	13.5
		2.98 ± 2.92	2.88 ± 2.86	3.23 ± 3.19	10.77 ± 9.77
		(208.31 ± 124.9)	(83.21 ± 38.49)	(291.62 ± 146)	
